# Hydrogen-bonding patterns in 5-fluoro­cytosine–melamine co-crystal (4/1)

**DOI:** 10.1107/S205698901600476X

**Published:** 2016-03-31

**Authors:** Marimuthu Mohana, Packianathan Thomas Muthiah, Liurukara D. Sanjeewa, Colin D. McMillen

**Affiliations:** a School of Chemistry, Bharathidasan University, Tiruchirappalli 620 024, Tamil Nadu, India; b Department of Chemistry, Clemson University, H. L. Hunter Laboratories, Clemson, SC 29634, USA

**Keywords:** crystal structure, 5-fluoro­cytosine, melamine, homosynthons, hydrogen bonding

## Abstract

The asymmetric unit of the title compound, 4C_4_H_4_FN_3_O·C_3_H_6_N_6_, comprises of two independent 5-fluoro­cytosine (5FC) mol­ecules (*A* and *B*) and one half-mol­ecule of melamine (*M*). The other half of the melamine mol­ecule is generated by a twofold axis. 5FC mol­ecules *A* and *B* are linked through two different homosynthons [*R*_2_^2^(8) ring motif]; one is formed *via* a pair of N—H⋯O hydrogen bonds and the second *via* a pair of N—H⋯N hydrogen bonds. In addition to this pairing, the O atoms of 5FC mol­ecules *A* and *B* inter­act with the N2 amino group on both sides of the melamine mol­ecule, forming a *DDAA* array of quadruple hydrogen bonds and generating a supra­molecular pattern. The 5FC (mol­ecules *A* and *B*) and two melamine mol­ecules inter­act *via* N—H⋯O, N—H⋯N and N—H⋯O, N—H⋯N, C—H⋯F hydrogen bonds forming *R*_6_^6^(24) and *R*_4_^4^(15) ring motifs. The crystal structure is further strengthened by C—H⋯F, C—F⋯π and π–π stacking inter­actions.

## Abstract 2

The asymmetric unit of the title compound comprises two independent 5-fluoro­cytosine mol­ecules and one half-mol­ecule of melamine. The 5-fluoro­cytosine mol­ecules are linked through two different homosynthons; one is formed *via* a pair of N—H⋯O hydrogen bonds and the second *via* a pair of N—H⋯N hydrogen bonds. The 5-fluoro­cytosine and melamine mol­ecules inter­act *via* N—H⋯O, N—H⋯N and N—H⋯O, N—H⋯N, C—H⋯F hydrogen bonds.

## Chemical context   

Pyrimidine derivatives are used in the treatment of anti­viral, anti­fungal, anti­tumor and cardiovascular diseases. 5-Fluoro­cytosine (5FC), a synthetic anti­mycotic compound, first synthesized in 1957 and widely used as an anti­tumor agent as a cytosine derivative (Tassel & Madoff, 1968 ▸; Benson & Nahata, 1988 ▸; Bennet, 1977 ▸; Polak & Scholer, 1980 ▸). It is active against fungal infection and was released in the year 1968 (Vermes *et al.*, 2000 ▸). It becomes active by deamination of 5FC into 5-fluoro­uracil by the enzyme cytosine deaminase (CD) and inhibits RNA and DNA synthesis (Larsen *et al.*, 2003 ▸; Mullen *et al.*, 1994 ▸; Morschhäuser, 2003 ▸). Melamine is a triazine derivative. It shows anti­tumor activity as well as biological activities such as anti­angiogenesis and anti­microbial effects. Triazine derivatives are useful synthons in supra­molecular chemistry. In particular, amino­triazines have been used for the formation of supra­molecular architectures using hydrogen bonds (Russell *et al.*, 1998 ▸; MacDonald & Whitesides, 1994 ▸; Whitesides *et al.*, 1991 ▸). The organic and inorganic salts develop well-defined non-covalent mol­ecular recognition *via* multiple hydrogen bonds by self assembly of components which contain a complementary array of hydrogen-bonding sites (Desiraju, 1989 ▸). The present work is focused on the supra­molecular hydrogen-bonding patterns exhibited by the co-crystal of 5-fluoro­cytosine with melamine.
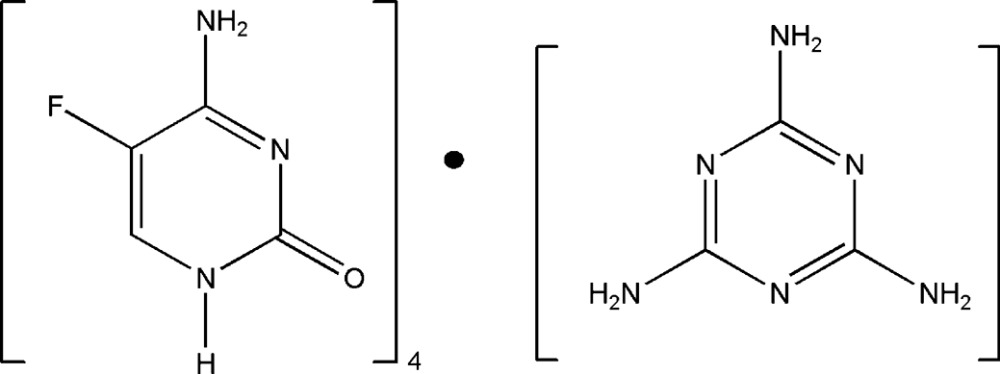


## Structural commentary   

**Figure 1:**
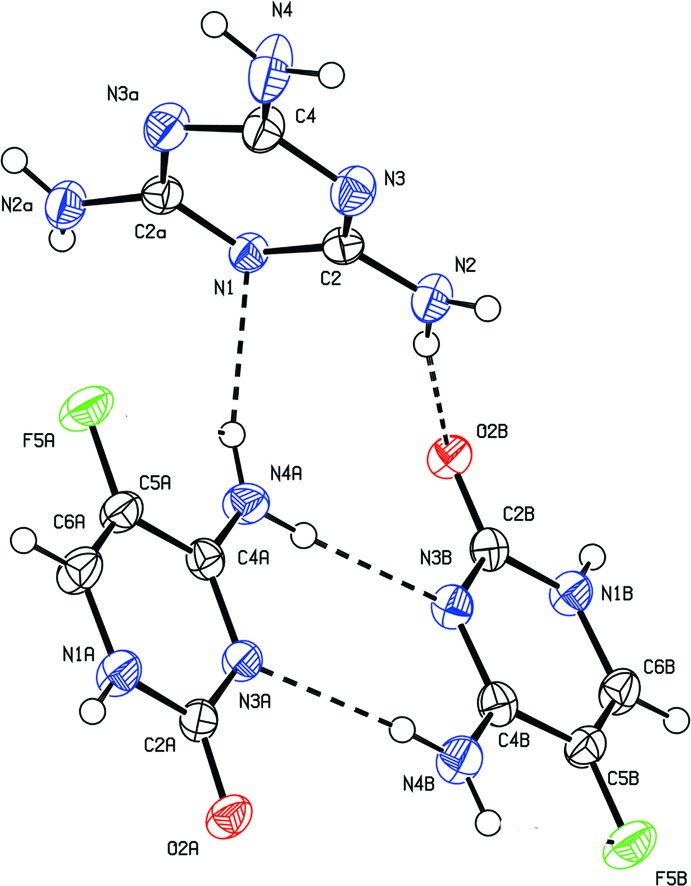
The asymmetric unit of the title compound, showing 30% probability displacement ellipsoids. Dashed lines indicate hydrogen bonds. Atoms with the suffix a are generated by the symmetry operation 1 − *x*, *y*, {\script{1\over 2}} − *z*.

The asymmetric unit comprises two independent 5-fluoro­cytosine (5FC) mol­ecules (*A* and *B*) and half a mol­ecule of melamine (*M*). The twofold axis of melamine coincides with the crystallographic twofold axis. An *ORTEP* view of the crystal structure is shown in Fig. 1 ▸. The values for the C—F bond distance in the two molecules [1.3491 (18) in 5FC *A* and 1.3492 (18) Å in 5FC *B* and the corresponding internal angles at the carbon-carrying fluorine atom [C2*A*—N3*A*—C4*A* = 119.96 (13) in 5FC *A* and C2*B*—N3*B*—C4*B* = 119.92 (13)° in 5FC *B*] agree with those reported in the literature (Louis *et al.*, 1982 ▸).

## Supra­molecular features   

**Figure 2:**
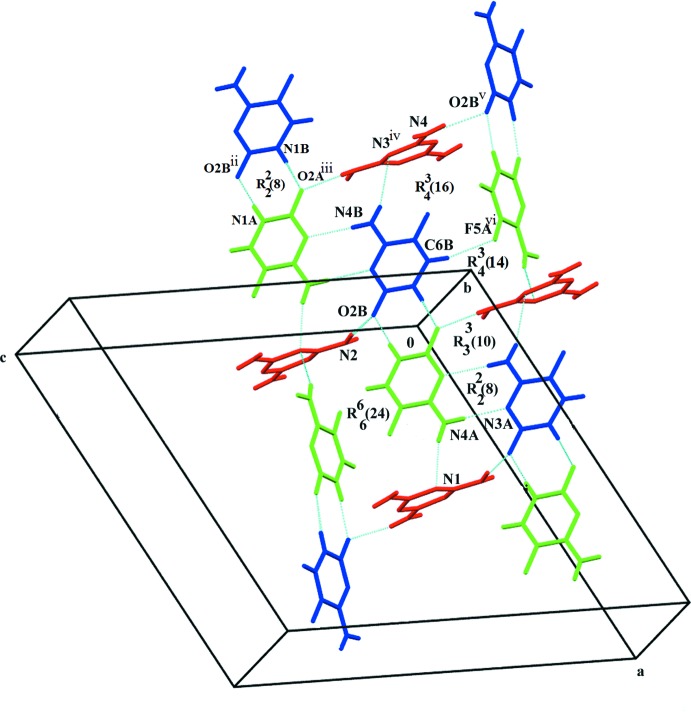
A view of the supra­molecular pattern involving two synthons formed by N—H⋯O hydrogen bonds. 5FC *A* mol­ecules are shown in green, 5FC *B* mol­ecules in blue and melamine in red. Blue dashed lines indicate hydrogen bonds. Symmetry codes are given in Table 1 ▸.

**Figure 3:**
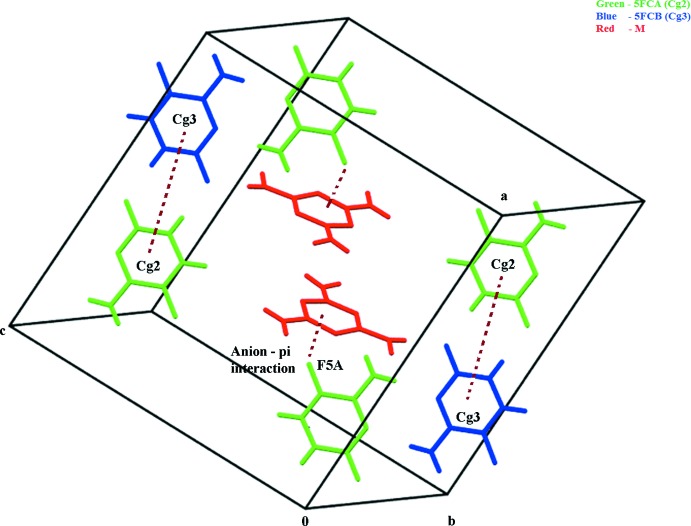
A view of C—F⋯π and aromatic π–π stacking inter­actions (dashed lines) between 5FC mol­ecules *A* and *B* and melamine.

Two different homosynthons are assembled *via* a pair of N—H⋯O and N—H⋯N hydrogen bonds (Table 1 ▸) to render a robust 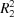
(8) ring motif. The first type of homosynthon is formed by the inter­action of the protonated N1 and O atoms of 5FC mol­ecules *A* and *B* through N—H⋯O hydrogen bonds. Another type of homosynthon is formed *via* the N4-amino and N3-pyrimidine ring nitro­gen atoms of the 5FC *A* and *B* mol­ecules through a pair of N—H⋯N hydrogen bonds (da Silva *et al.*, 2013 ▸; Tutughamiarso *et al.*, 2012 ▸). The melamine mol­ecule and 5FC (mol­ecules *A* and *B*) are involved in the generation of a quadruple hydrogen-bonded *DDAA* array having a fused-ring sequence of 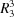
(10), 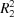
(8) and 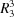
(10). The 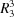
(10) motif is formed on both sides *via* N—H⋯O and N—H⋯N hydrogen bonds. These quadruple arrays are further linked by three large ring motifs: 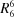
(24), 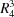
(16) and 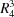
(14). The 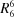
(24) ring motifs are formed by the inter­action of two 5FC *A* mol­ecules, two 5FC *B* mol­ecules and two melamine mol­ecules through several N—H⋯O and N—H⋯N hydrogen bonds, generating a hexa­meric supermolecule. The 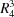
(16) ring motif links the one 5FC *A* mol­ecule, two 5FC *B* mol­ecules and one melamine mol­ecule through N—H⋯O, N—H⋯N and C—H⋯F hydrogen bonds, generating a tetra­meric supermolecule. Similarly, the 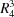
(14) ring motifs are formed by the inter­action of two 5FC *A* mol­ecules, one 5FC *B* mol­ecule and one melamine mol­ecule through N—H⋯O, N—H⋯N and C—H⋯F hydrogen bonds, generating another tetra­meric supermolecule. The association of these 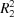
(8), *DDAA* array and 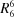
(24), 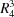
(16) and 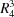
(14) motifs leads to the formation of supra­molecular patterns (Fig. 2 ▸). The crystal structure is also stabilized by weak C—H⋯F hydrogen bonds and π–π stacking inter­actions between 5FC *A* and *B* mol­ecules with an inter­planar distance of 3.475 (6) Å, centroid-to-centroid distance of 3.6875 (11) Å, and slip angle of 19.52°. The crystal structure is further strengthened by a C—F⋯π inter­action [3.4541 (14) Å] between 5-fluoro­cytosinium mol­ecule *A* and the melamine mol­ecule (Fig. 3 ▸).

In this co-crystal, 5FC mol­ecules *A* and *B* form two types of homosynthons (two types of base pairing) while the melamine mol­ecule inter­acts with them *via* N—H⋯O and N—H⋯N hydrogen bonds, generating the supra­molecular architecture.

## Database survey   

The crystal structure of 5-fluoro­cytosine monohydrate (Louis *et al.*, 1982 ▸; Portalone & Colapietro, 2006 ▸; Portalone, 2011 ▸), polymorphs (Hulme & Tocher, 2006 ▸; Tutughamiarso *et al.*, 2009 ▸), salts (Perumalla *et al.*, 2013*a* ▸,*b* ▸) and co-crystals (Tutughamiarso *et al.*, 2012 ▸; Da Silva *et al.*, 2013 ▸) have been reported in the literature. From our laboratory, 5-fluoro­cytosinium salicylate (Prabakaran *et al.*, 2001 ▸) and 5-fluoro­cytosinium 3-hy­droxy­picolinate (Karthikeyan *et al.*, 2014 ▸) have been reported. Various salts, co-crystals and metal complexes of melamine have also been reported (Janczak & Perpétuo, 2001*a* ▸,*b* ▸, 2002 ▸, 2004 ▸; Perpétuo *et al.*, 2005 ▸; Zerkowski & Whitesides, 1994 ▸; Wang *et al.*, 2007 ▸).

## Synthesis and crystallization   

Hot aqueous solutions of 5-fluoro­cytosine (32 mg) and melamine (31 mg) were mixed in a 1:1 molar ratio. The resulting solution was warmed to 353 K using a water bath for half an hour and kept at room temperature for crystallization. After one week, colourless crystals were obtained.

## Refinement details   

Crystal data, data collection and structure refinement details are summarized in Table 2 ▸. The hydrogen atoms of amino (N2, N4, N4*A*, N4*B*) groups were located in a difference Fourier map and refined freely. The other hydrogen atoms were positioned geometrically (C—H = 0.95, N—H = 0.88 Å) and were refined using a riding model with *U*_iso_(H) = 1.2*U*_eq_(parent atom).

## Supplementary Material

Crystal structure: contains datablock(s) global, I. DOI: 10.1107/S205698901600476X/hg5470sup1.cif

Structure factors: contains datablock(s) I. DOI: 10.1107/S205698901600476X/hg5470Isup2.hkl

Supporting information file. DOI: 10.1107/S205698901600476X/hg5470Isup3.cml

CCDC reference: 1469709

Additional supporting information:  crystallographic information; 3D view; checkCIF report

## Figures and Tables

**Table 1:** Hydrogen-bond geometry (Å, °)

*D*—H⋯*A*	*D*—H	H⋯*A*	*D*⋯*A*	*D*—H⋯*A*
N4*A*—H4*A*1⋯F5*A*	0.86 (2)	2.47 (2)	2.7560 (18)	100.0 (18)
N4*A*—H4*A*1⋯N1	0.86 (2)	2.23 (2)	3.0664 (18)	164 (2)
N1*A*—H1*A*⋯O2*B* ^ii^	0.88	1.90	2.773 (2)	173
N1*B*—H1*B*⋯O2*A* ^iii^	0.88	1.88	2.7545 (19)	175
N4*A*—H4*A*2⋯N3*B*	0.91 (2)	2.10 (2)	2.992 (2)	169 (2)
N2—H2*A*⋯O2*B*	0.89 (2)	2.10 (2)	2.9689 (19)	167.6 (18)
N2—H2*B*⋯O2*A* ^iv^	0.84 (2)	2.15 (2)	2.8949 (19)	149 (2)
N4*B*—H4*B*1⋯N3*A*	0.88 (2)	2.20 (2)	3.060 (2)	169 (2)
N4*B*—H4*B*2⋯F5*B*	0.86 (2)	2.42 (2)	2.7459 (19)	103 (2)
N4*B*—H4*B*2⋯N3^iv^	0.86 (2)	2.53 (2)	3.360 (2)	162 (2)
N4—H4*A*⋯O2*B* ^v^	0.89 (2)	2.09 (2)	2.9600 (15)	165 (2)
C6*B*—H6*B*⋯F5*A* ^vi^	0.95	2.43	3.2444 (19)	143

Symmetry codes: (ii) 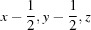; (iii) 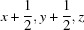; (iv) 
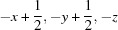
; (v) 
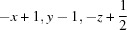
; (vi) 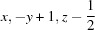.

**Table 2:** Experimental details

Crystal data	
Chemical formula	4C_4_H_4_FN_3_O·C_3_H_6_N_6_
*M*_r_	642.55
Crystal system, space group	Monoclinic, *C*2/*c*
Temperature (K)	200
*a*, *b*, *c* (Å)	18.343 (4), 7.9591 (16), 19.680 (4)
β (°)	114.65 (3)
*V* (Å^3^)	2611.3 (11)
*Z*	4
Radiation type	Mo *K*α
μ (mm^−1^)	0.14
Crystal size (mm)	0.20 × 0.20 × 0.20

Data collection	
Diffractometer	Rigaku AFC–8S
Absorption correction	Multi-scan (*CrystalClear*; Rigaku/MSC, 2008 ▸)
*T*_min_, *T*_max_	0.972, 0.972
No. of measured, independent and observed [*I* > 2σ(*I*)] reflections	10071, 2564, 2362
*R*_int_	0.019
(sin θ/λ)_max_ (Å^−1^)	0.617

Refinement	
*R*[*F* ^2^ > 2σ(*F* ^2^)], *wR*(*F* ^2^), *S*	0.045, 0.123, 1.07
No. of reflections	2564
No. of parameters	233
H-atom treatment	H atoms treated by a mixture of independent and constrained refinement
Δρ_max_, Δρ_min_ (e Å^−3^)	0.44, −0.34

Computer programs: *CrystalClear* (Rigaku/MSC, 2008 ▸), *SHELXS97* and *SHELXL97* (Sheldrick, 2008 ▸), *PLATON* (Spek, 2009 ▸), *Mercury* (Macrae *et al.*, 2008 ▸), *POV-RAY* (Cason, 2004 ▸) and *publCIF* (Westrip, 2010 ▸).

## supplementary crystallographic information

### Crystal data

**Table:**

4(C_4_H_4_FN_3_O)·C_3_H_6_N_6_	*F*(000) = 1320
*M**_r_* = 642.55	*D*_x_ = 1.634 Mg m^−^^3^
Monoclinic, *C*2/*c*	Mo *K*α radiation, λ = 0.71073 Å
Hall symbol: -C 2yc	Cell parameters from 2564 reflections
*a* = 18.343 (4) Å	θ = 2.4–26.0°
*b* = 7.9591 (16) Å	µ = 0.14 mm^−^^1^
*c* = 19.680 (4) Å	*T* = 200 K
β = 114.65 (3)°	Prism, colorless
*V* = 2611.3 (11) Å^3^	0.20 × 0.20 × 0.20 mm
*Z* = 4	

### Data collection

**Table:**

Rigaku AFC–8S diffractometer	2564 independent reflections
Radiation source: fine focus sealed tube	2362 reflections with *I* > 2σ(*I*)
Graphite monochromator	*R*_int_ = 0.019
Detector resolution: 14.6199 pixels mm^-1^	θ_max_ = 26.0°, θ_min_ = 2.4°
ω scans	*h* = −22→18
Absorption correction: multi-scan (*CrystalClear*; Rigaku/MSC, 2008)	*k* = −9→9
*T*_min_ = 0.972, *T*_max_ = 0.972	*l* = −24→24
10071 measured reflections	

### Refinement

**Table:**

Refinement on *F*^2^	Primary atom site location: structure-invariant direct methods
Least-squares matrix: full	Secondary atom site location: difference Fourier map
*R*[*F*^2^ > 2σ(*F*^2^)] = 0.045	Hydrogen site location: inferred from neighbouring sites
*wR*(*F*^2^) = 0.123	H atoms treated by a mixture of independent and constrained refinement
*S* = 1.07	*w* = 1/[σ^2^(*F*_o_^2^) + (0.0741*P*)^2^ + 1.8751*P*] where *P* = (*F*_o_^2^ + 2*F*_c_^2^)/3
2564 reflections	(Δ/σ)_max_ < 0.001
233 parameters	Δρ_max_ = 0.44 e Å^−^^3^
0 restraints	Δρ_min_ = −0.34 e Å^−^^3^

### Special details

**Table:**

Geometry. Bond distances, angles *etc*. have been calculated using the rounded fractional coordinates. All su's are estimated from the variances of the (full) variance-covariance matrix. The cell e.s.d.'s are taken into account in the estimation of distances, angles and torsion angles
Refinement. Refinement on *F*^2^ for ALL reflections except those flagged by the user for potential systematic errors. Weighted *R*-factors *wR* and all goodnesses of fit *S* are based on *F*^2^, conventional *R*-factors *R* are based on *F*, with *F* set to zero for negative *F*^2^. The observed criterion of *F*^2^ > σ(*F*^2^) is used only for calculating -*R*-factor-obs *etc*. and is not relevant to the choice of reflections for refinement. *R*-factors based on *F*^2^ are statistically about twice as large as those based on *F*, and *R*-factors based on ALL data will be even larger.

### Fractional atomic coordinates and isotropic or equivalent isotropic displacement parameters (Å^2^)

**Table:**

	*x*	*y*	*z*	*U*_iso_*/*U*_eq_	
N1	0.50000	0.1866 (2)	0.25000	0.0244 (5)	
N2	0.48606 (8)	0.18336 (19)	0.12847 (8)	0.0326 (4)	
N3	0.48592 (8)	−0.07579 (17)	0.18444 (7)	0.0342 (4)	
N4	0.50000	−0.3280 (3)	0.25000	0.0501 (8)	
C2	0.49100 (8)	0.09524 (18)	0.18883 (7)	0.0248 (4)	
C4	0.50000	−0.1589 (3)	0.25000	0.0300 (6)	
F5A	0.32327 (5)	0.12206 (14)	0.26116 (5)	0.0420 (3)	
O2A	0.06416 (6)	0.34719 (15)	0.03132 (6)	0.0326 (3)	
N1A	0.11896 (8)	0.17904 (17)	0.13300 (7)	0.0315 (4)	
N3A	0.19980 (7)	0.34775 (15)	0.09362 (7)	0.0252 (3)	
N4A	0.33640 (8)	0.35220 (17)	0.16222 (8)	0.0299 (4)	
C2A	0.12590 (8)	0.29396 (19)	0.08380 (8)	0.0253 (4)	
C4A	0.26467 (8)	0.29295 (18)	0.15183 (8)	0.0243 (4)	
C5A	0.25656 (9)	0.17587 (19)	0.20310 (8)	0.0286 (4)	
C6A	0.18349 (9)	0.1215 (2)	0.19258 (9)	0.0332 (5)	
F5B	0.21480 (6)	0.72438 (14)	−0.15032 (5)	0.0419 (3)	
O2B	0.46711 (6)	0.54980 (13)	0.09792 (6)	0.0280 (3)	
N1B	0.41539 (7)	0.70320 (16)	−0.00875 (7)	0.0278 (3)	
N3B	0.33298 (7)	0.53026 (15)	0.02705 (7)	0.0261 (3)	
N4B	0.19773 (8)	0.51382 (18)	−0.04738 (8)	0.0328 (4)	
C2B	0.40666 (8)	0.59203 (17)	0.04058 (8)	0.0240 (4)	
C4B	0.26965 (9)	0.57389 (18)	−0.03540 (8)	0.0259 (4)	
C5B	0.27992 (9)	0.68402 (19)	−0.08772 (8)	0.0285 (4)	
C6B	0.35239 (9)	0.74902 (19)	−0.07308 (8)	0.0298 (4)	
H2A	0.4807 (12)	0.294 (3)	0.1263 (11)	0.037 (5)*	
H2B	0.4701 (12)	0.135 (3)	0.0868 (12)	0.042 (5)*	
H4A	0.5134 (13)	−0.381 (3)	0.2935 (11)	0.048 (6)*	
H4A1	0.3799 (13)	0.308 (3)	0.1947 (11)	0.040 (5)*	
H1A	0.07100	0.14180	0.12550	0.0380*	
H4A2	0.3380 (13)	0.418 (3)	0.1253 (13)	0.049 (6)*	
H6A	0.17710	0.04390	0.22640	0.0400*	
H1B	0.46300	0.74610	0.00150	0.0330*	
H4B1	0.1965 (13)	0.453 (3)	−0.0107 (12)	0.047 (6)*	
H4B2	0.1569 (13)	0.543 (3)	−0.0874 (13)	0.045 (6)*	
H6B	0.35960	0.82540	−0.10690	0.0360*	

### Atomic displacement parameters (Å^2^)

**Table:**

	*U*^11^	*U*^22^	*U*^33^	*U*^12^	*U*^13^	*U*^23^
N1	0.0248 (8)	0.0253 (8)	0.0219 (8)	0.0000	0.0085 (7)	0.0000
N2	0.0389 (8)	0.0345 (8)	0.0244 (7)	−0.0006 (6)	0.0133 (6)	0.0007 (5)
N3	0.0418 (8)	0.0307 (7)	0.0290 (7)	−0.0010 (5)	0.0138 (6)	−0.0013 (5)
N4	0.098 (2)	0.0262 (10)	0.0346 (11)	0.0000	0.0362 (13)	0.0000
C2	0.0196 (6)	0.0293 (7)	0.0231 (7)	0.0011 (5)	0.0064 (5)	−0.0001 (5)
C4	0.0387 (11)	0.0260 (10)	0.0245 (10)	0.0000	0.0124 (9)	0.0000
F5A	0.0276 (5)	0.0583 (7)	0.0320 (5)	0.0010 (4)	0.0043 (4)	0.0153 (4)
O2A	0.0229 (5)	0.0448 (7)	0.0268 (5)	−0.0027 (4)	0.0070 (4)	0.0085 (5)
N1A	0.0245 (6)	0.0405 (7)	0.0289 (7)	−0.0059 (5)	0.0106 (5)	0.0071 (5)
N3A	0.0222 (6)	0.0289 (6)	0.0244 (6)	−0.0020 (5)	0.0096 (5)	0.0018 (5)
N4A	0.0216 (6)	0.0349 (7)	0.0318 (7)	−0.0005 (5)	0.0099 (6)	0.0054 (5)
C2A	0.0245 (7)	0.0299 (7)	0.0219 (7)	−0.0018 (5)	0.0101 (6)	−0.0004 (5)
C4A	0.0250 (7)	0.0249 (7)	0.0239 (7)	−0.0008 (5)	0.0112 (6)	−0.0031 (5)
C5A	0.0261 (7)	0.0334 (8)	0.0226 (7)	0.0005 (6)	0.0065 (6)	0.0033 (6)
C6A	0.0312 (8)	0.0387 (8)	0.0283 (8)	−0.0037 (6)	0.0111 (6)	0.0090 (6)
F5B	0.0300 (5)	0.0569 (6)	0.0302 (5)	−0.0019 (4)	0.0041 (4)	0.0115 (4)
O2B	0.0250 (5)	0.0298 (5)	0.0269 (5)	−0.0040 (4)	0.0085 (4)	0.0023 (4)
N1B	0.0246 (6)	0.0310 (6)	0.0282 (6)	−0.0051 (5)	0.0114 (5)	0.0032 (5)
N3B	0.0245 (6)	0.0270 (6)	0.0279 (6)	−0.0055 (5)	0.0119 (5)	−0.0003 (5)
N4B	0.0258 (7)	0.0408 (8)	0.0294 (7)	−0.0066 (5)	0.0092 (6)	0.0010 (6)
C2B	0.0267 (7)	0.0226 (6)	0.0241 (7)	−0.0030 (5)	0.0119 (6)	−0.0030 (5)
C4B	0.0274 (7)	0.0254 (7)	0.0259 (7)	−0.0032 (5)	0.0122 (6)	−0.0045 (5)
C5B	0.0267 (7)	0.0341 (8)	0.0217 (7)	0.0007 (6)	0.0072 (6)	0.0014 (6)
C6B	0.0320 (8)	0.0328 (8)	0.0257 (7)	−0.0021 (6)	0.0130 (6)	0.0040 (6)

### Geometric parameters (Å, º)

**Table:**

F5A—C5A	1.3491 (18)	N4A—C4A	1.331 (2)
F5B—C5B	1.3492 (18)	N1A—H1A	0.8800
O2A—C2A	1.2462 (19)	N4A—H4A2	0.91 (2)
O2B—C2B	1.2539 (19)	N4A—H4A1	0.86 (2)
N1—C2	1.3563 (16)	N1B—C2B	1.3714 (19)
N1—C2^i^	1.3563 (16)	N1B—C6B	1.361 (2)
N2—C2	1.350 (2)	N3B—C4B	1.338 (2)
N3—C2	1.365 (2)	N3B—C2B	1.356 (2)
N3—C4	1.3751 (18)	N4B—C4B	1.329 (2)
N4—C4	1.346 (3)	N1B—H1B	0.8800
N2—H2A	0.89 (2)	N4B—H4B1	0.88 (2)
N2—H2B	0.84 (2)	N4B—H4B2	0.86 (2)
N4—H4A	0.89 (2)	C4A—C5A	1.426 (2)
N4—H4A^i^	0.89 (2)	C5A—C6A	1.340 (3)
N1A—C2A	1.376 (2)	C6A—H6A	0.9500
N1A—C6A	1.352 (2)	C4B—C5B	1.423 (2)
N3A—C4A	1.335 (2)	C5B—C6B	1.341 (2)
N3A—C2A	1.356 (2)	C6B—H6B	0.9500
			
F5A···C2^i^	3.134 (2)	C4B···C4B^ix^	3.340 (2)
F5A···C6B^ii^	3.2444 (19)	C5A···C5B^v^	3.541 (2)
F5A···N4A	2.7560 (18)	C5B···C6A^v^	3.432 (2)
F5B···N4B	2.7459 (19)	C5B···C5A^v^	3.541 (2)
F5B···C6A^iii^	3.148 (2)	C5B···C4B^ix^	3.500 (2)
F5A···H4A1	2.47 (2)	C6A···F5B^ii^	3.148 (2)
F5A···H6B^ii^	2.4300	C6A···C5B^v^	3.432 (2)
F5B···H4B2	2.42 (2)	C6B···N3A^ix^	3.325 (2)
O2A···N1B^iv^	2.7545 (19)	C6B···F5A^iii^	3.2444 (19)
O2A···N2^v^	2.8949 (19)	C6B···N4B^ix^	3.442 (2)
O2B···N4^vi^	2.9600 (15)	C2···H4A1	2.69 (2)
O2B···N2	2.9689 (19)	C2···H4A1^i^	3.03 (2)
O2B···N4^vii^	2.9600 (15)	C2···H4B2^v^	2.84 (2)
O2B···N1A^viii^	2.773 (2)	C2A···H4B1	2.96 (2)
O2A···H1B^iv^	1.8800	C2A···H6B^ix^	3.0600
O2A···H2B^v^	2.15 (2)	C2A···H1B^iv^	2.7700
O2B···H4A^vii^	2.09 (2)	C2B···H1A^viii^	2.8000
O2B···H4A2	2.84 (3)	C2B···H4A^vii^	2.98 (2)
O2B···H1A^viii^	1.9000	C2B···H4A2	2.84 (2)
O2B···H2A	2.10 (2)	C2B···H2A	2.90 (2)
N1···N4A	3.0664 (18)	C4A···H6A^xii^	2.9600
N1···N4A^i^	3.0664 (18)	H4A1···C2	2.69 (2)
N1A···O2B^iv^	2.773 (2)	H4A1···N1	2.23 (2)
N1B···O2A^viii^	2.7545 (19)	H4A1···N2	2.93 (2)
N2···O2B	2.9689 (19)	H4A1···N1	2.23 (2)
N2···O2A^v^	2.8949 (19)	H4A1···F5A	2.47 (2)
N3A···N4B	3.060 (2)	H4A1···C2^i^	3.03 (2)
N3A···C6B^ix^	3.325 (2)	H1A···C2B^iv^	2.8000
N3B···N4A	2.992 (2)	H1A···H1B^iv^	2.5600
N4···O2B^x^	2.9600 (15)	H1A···O2B^iv^	1.9000
N4···O2B^xi^	2.9600 (15)	H1B···C2A^viii^	2.7700
N4A···N1	3.0664 (18)	H1B···O2A^viii^	1.8800
N4A···N1	3.0664 (18)	H1B···H1A^viii^	2.5600
N4A···C2	3.356 (2)	H4A2···O2B	2.84 (3)
N4A···N3B	2.992 (2)	H4A2···N3B	2.10 (2)
N4A···F5A	2.7560 (18)	H4A2···C2B	2.84 (2)
N4B···C4A^v^	3.442 (2)	H2A···C2B	2.90 (2)
N4B···N3A	3.060 (2)	H2A···O2B	2.10 (2)
N4B···F5B	2.7459 (19)	H2B···O2A^v^	2.15 (2)
N4B···C6B^ix^	3.442 (2)	H4B1···N3A	2.20 (2)
N1···H4A1	2.23 (2)	H4B1···C2A	2.96 (2)
N1···H4A1^i^	2.23 (2)	H4B2···F5B	2.42 (2)
N2···H4A1	2.93 (2)	H4B2···N3^v^	2.53 (2)
N3···H4B2^v^	2.53 (2)	H4B2···C2^v^	2.84 (2)
N3A···H6B^ix^	2.8700	H4A···O2B^x^	2.09 (2)
N3A···H4B1	2.20 (2)	H4A···C2B^x^	2.98 (2)
N3B···H4A2	2.10 (2)	H6A···N4A^xiii^	2.7700
N4A···H6A^xii^	2.7700	H6A···C4A^xiii^	2.9600
C2···N4A	3.356 (2)	H6B···F5A^iii^	2.4300
C2···F5A^i^	3.134 (2)	H6B···N3A^ix^	2.8700
C4A···N4B^v^	3.442 (2)	H6B···C2A^ix^	3.0600
C4B···C5B^ix^	3.500 (2)		
			
C2—N1—C2^i^	115.16 (14)	N3—C4—N3^i^	122.49 (19)
C2—N3—C4	116.06 (14)	N3—C4—N4	118.75 (11)
C2—N2—H2B	119.3 (16)	O2A—C2A—N1A	119.37 (15)
H2A—N2—H2B	115 (2)	N1A—C2A—N3A	119.33 (14)
C2—N2—H2A	121.7 (13)	O2A—C2A—N3A	121.30 (14)
C4—N4—H4A	118.2 (15)	N3A—C4A—N4A	119.11 (14)
H4A—N4—H4A^i^	124 (2)	N3A—C4A—C5A	120.14 (15)
C4—N4—H4A^i^	118.2 (15)	N4A—C4A—C5A	120.73 (14)
C2A—N1A—C6A	122.07 (15)	F5A—C5A—C6A	121.59 (14)
C2A—N3A—C4A	119.96 (13)	F5A—C5A—C4A	118.77 (15)
C6A—N1A—H1A	119.00	C4A—C5A—C6A	119.64 (15)
C2A—N1A—H1A	119.00	N1A—C6A—C5A	118.83 (15)
C4A—N4A—H4A1	121.3 (16)	N1A—C6A—H6A	121.00
C4A—N4A—H4A2	116.3 (16)	C5A—C6A—H6A	121.00
H4A1—N4A—H4A2	120 (2)	O2B—C2B—N3B	120.96 (14)
C2B—N1B—C6B	121.81 (14)	N1B—C2B—N3B	119.73 (14)
C2B—N3B—C4B	119.92 (13)	O2B—C2B—N1B	119.31 (14)
C2B—N1B—H1B	119.00	N3B—C4B—C5B	119.89 (16)
C6B—N1B—H1B	119.00	N4B—C4B—C5B	120.96 (15)
C4B—N4B—H4B2	119.0 (17)	N3B—C4B—N4B	119.15 (14)
H4B1—N4B—H4B2	126 (2)	C4B—C5B—C6B	120.04 (14)
C4B—N4B—H4B1	114.6 (16)	F5B—C5B—C4B	118.25 (15)
N2—C2—N3	119.01 (13)	F5B—C5B—C6B	121.68 (14)
N1—C2—N2	116.19 (14)	N1B—C6B—C5B	118.54 (14)
N1—C2—N3	124.81 (13)	N1B—C6B—H6B	121.00
N3^i^—C4—N4	118.75 (11)	C5B—C6B—H6B	121.00
			
C2^i^—N1—C2—N2	−176.73 (13)	C4B—N3B—C2B—O2B	−178.38 (14)
C2^i^—N1—C2—N3	3.98 (19)	C2B—N3B—C4B—N4B	−179.21 (14)
C4—N3—C2—N1	−7.5 (2)	C2B—N3B—C4B—C5B	0.5 (2)
C4—N3—C2—N2	173.24 (13)	C4B—N3B—C2B—N1B	2.1 (2)
C2—N3—C4—N4	−176.55 (11)	N3A—C4A—C5A—F5A	179.63 (13)
C2—N3—C4—N3^i^	3.45 (18)	N3A—C4A—C5A—C6A	−0.1 (2)
C6A—N1A—C2A—O2A	−177.48 (15)	N4A—C4A—C5A—F5A	−2.1 (2)
C6A—N1A—C2A—N3A	2.2 (2)	N4A—C4A—C5A—C6A	178.19 (15)
C2A—N1A—C6A—C5A	−1.3 (2)	F5A—C5A—C6A—N1A	−179.48 (14)
C4A—N3A—C2A—O2A	177.67 (14)	C4A—C5A—C6A—N1A	0.3 (2)
C4A—N3A—C2A—N1A	−2.0 (2)	N3B—C4B—C5B—F5B	179.56 (14)
C2A—N3A—C4A—N4A	−177.35 (14)	N3B—C4B—C5B—C6B	−2.6 (2)
C2A—N3A—C4A—C5A	1.0 (2)	N4B—C4B—C5B—F5B	−0.8 (2)
C6B—N1B—C2B—N3B	−2.7 (2)	N4B—C4B—C5B—C6B	177.13 (15)
C2B—N1B—C6B—C5B	0.6 (2)	F5B—C5B—C6B—N1B	179.78 (14)
C6B—N1B—C2B—O2B	177.75 (14)	C4B—C5B—C6B—N1B	2.0 (2)

Symmetry codes: (i) −*x*+1, *y*, −*z*+1/2; (ii) *x*, −*y*+1, *z*+1/2; (iii) *x*, −*y*+1, *z*−1/2; (iv) *x*−1/2, *y*−1/2, *z*; (v) −*x*+1/2, −*y*+1/2, −*z*; (vi) *x*, *y*+1, *z*; (vii) −*x*+1, *y*+1, −*z*+1/2; (viii) *x*+1/2, *y*+1/2, *z*; (ix) −*x*+1/2, −*y*+3/2, −*z*; (x) −*x*+1, *y*−1, −*z*+1/2; (xi) *x*, *y*−1, *z*; (xii) −*x*+1/2, *y*+1/2, −*z*+1/2; (xiii) −*x*+1/2, *y*−1/2, −*z*+1/2.

### Hydrogen-bond geometry (Å, º)

**Table:**

*D*—H···*A*	*D*—H	H···*A*	*D*···*A*	*D*—H···*A*
N4*A*—H4*A*1···F5*A*	0.86 (2)	2.47 (2)	2.7560 (18)	100.0 (18)
N4*A*—H4*A*1···N1	0.86 (2)	2.23 (2)	3.0664 (18)	164 (2)
N1*A*—H1*A*···O2*B*^iv^	0.88	1.90	2.773 (2)	173
N1*B*—H1*B*···O2*A*^viii^	0.88	1.88	2.7545 (19)	175
N4*A*—H4*A*2···N3*B*	0.91 (2)	2.10 (2)	2.992 (2)	169 (2)
N2—H2*A*···O2*B*	0.89 (2)	2.10 (2)	2.9689 (19)	167.6 (18)
N2—H2*B*···O2*A*^v^	0.84 (2)	2.15 (2)	2.8949 (19)	149 (2)
N4*B*—H4*B*1···N3*A*	0.88 (2)	2.20 (2)	3.060 (2)	169 (2)
N4*B*—H4*B*2···F5*B*	0.86 (2)	2.42 (2)	2.7459 (19)	103 (2)
N4*B*—H4*B*2···N3^v^	0.86 (2)	2.53 (2)	3.360 (2)	162 (2)
N4—H4*A*···O2*B*^x^	0.89 (2)	2.09 (2)	2.9600 (15)	165 (2)
C6*B*—H6*B*···F5*A*^iii^	0.95	2.43	3.2444 (19)	143

Symmetry codes: (iii) *x*, −*y*+1, *z*−1/2; (iv) *x*−1/2, *y*−1/2, *z*; (v) −*x*+1/2, −*y*+1/2, −*z*; (viii) *x*+1/2, *y*+1/2, *z*; (x) −*x*+1, *y*−1, −*z*+1/2.
